# Supplementary material for: Spiking neurons as predictive controllers of linear systems
Source: PLoS Comput Biol. 2026 Jul 9;22(7):e1014432. doi: 10.1371/journal.pcbi.1014432 (PMC13384403; doi:10.1371/journal.pcbi.1014432)
Supplement: S1 Appendix — In the supporting S1 Appendix, we address several aspects of the proposed spiking control framework that complement the main results. We demonstrate how control performance is affected when the assumption of asynchronous firing is relaxed, and show that a spike-threshold adaptation mechanism restores sparse activity and stable control under delayed or synchronous firing conditions. We further discuss alternative measures of control energy, including the total work performed on the controlled system. Finally, we investigate the robustness of the network to voltage noise over a wide range of amplitudes, characterizing the transition from sparse and accurate control to high-activity regimes in which noise degrades control performance. Supplementary figures provide illustrative examples of these effects and their implications for spiking control networks. (PDF) [file pcbi.1014432.s001.pdf]

S1 Appendix - Supporting information for Spiking neurons as predictive controllers of linear systems

Paolo Umberto Agliati

Jun 2026

Control with synchronous firing

As mentioned in Results and Materials and Methods, we employ an asynchronous firing rule in all the examples of this paper, to showcase the simplest case of our spiking control. Of course, the assumption that only one neuron fires at any one time is generally not realistic. Furthermore, regardless of the level of biological detail in the network, asynchronous firing could not work for certain control tasks. For instance, if there exists any time delay between the system reaching a state and the neurons detecting such state, then multiple neurons would fire in response to the same loss comparison, and the system would be over-controlled, eventually missing the target. In a control problem with enough neurons and enough dimensions (where the mapping of the control signals greatly overlap), this would result in most of the neurons firing almost all the time, completely losing the sparsity of the spiking activity, as shown in Fig AA in S1 Appendix. This happens because our network is fully local: neurons do not have access to the state of other neurons in the network via their connectivity, and thus compute their spiking decision independently from each other. Important to note that for simulations with zero delays that use very small timesteps, the asynchronous firing regime is naturally maintained, but for larger timesteps or in the presence of delays this is no longer feasible. The derivation of our network accounts for these issues and proposes an architecture that does not require impositions on the neuron's firing mechanism, while still retaining the local properties. To do so, we introduce a spike-threshold adaptation term (see Materials and Methods), which scales the threshold of each neuron based on its past activity. As shown in Fig AB in S1 Appendix, employing a spike-threshold adaptation mechanism allows to recover control performance and a sparse spiking regime in case delays are present in the system (or in general, in the absence of asynchronous firing).

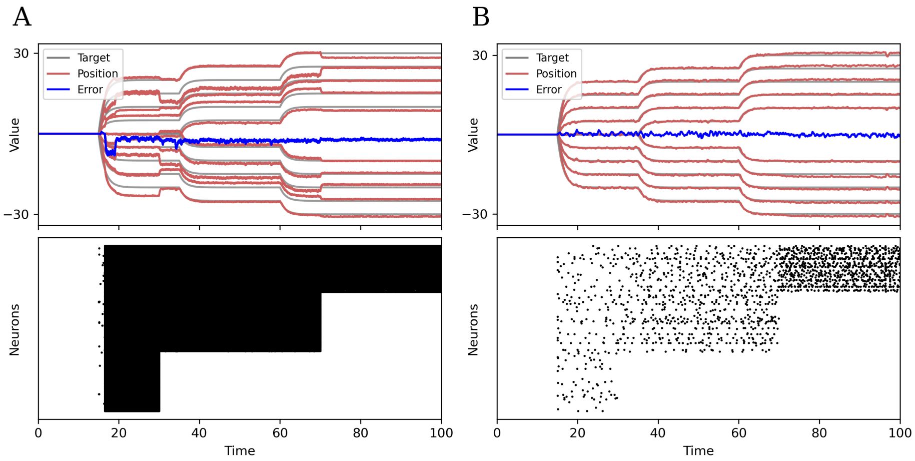

Figure A: Control examples without asynchronous firing. A: Activity (top panel) and spike raster (lower panel) of a network of 500 neurons controlling a coupled SMD system of 10 oscillating masses. The example is identical to the one presented in Fig 8, but the network showcased here does not operate with an asynchronous firing rule. In this case, more neurons are allowed to spike at the same timestep, and no spike-threshold adaptation mechanism is employed. As a result, the control performance is clearly affected, and all neurons are spiking at any given time. The local spiking rule of each neuron cannot account for this scenario, since no neuron has access to the state of other neurons in the network. B: Activity (top panel) and spike raster (lower panel) of a network of 500 neurons controlling a coupled SMD system of 10 oscillating masses. The example is identical to the one presented in Fig 8, but the network showcased here does not operate with an asynchronous firing rule. In this case, a spike-threshold adaptation mechanism is employed, so that neurons that had higher activity will be less likely to fire. As shown, this adaptive mechanism allows us to retain the local spiking rule without having an over-spiking regime, and without affecting the control performance.

Energy measures: work done

When considering the energy usage of our algorithm on the controlled plant (see Fig 6), we refer to the integrated acceleration onto the system. This quantity is very easily defined and interpreted, as it is only constituted by the change in velocity that occurs when a spike is emitted and mapped onto the system via the matrix $\mathbf{B}$. The energy we consider is thus the sum of the scaled spikes at their spiking times during the whole length of the simulation, T_{end}. The matrix $\mathbf{B}$ has zeros in all the rows and number b_i for the row of neuron i, to map each spike j onto the system.

$$W = \sum_{i=1}^{N} \sum_{j=1}^{K_i} \int_0^{T_{\text{end}}} b_i \delta(t - t_j^i)\, \mathrm{d}t$$

where N is the number of neurons and K_i is the amount of spikes fired by neuron i, such that we sum over all neurons and all spikes. Another principled way to consider the effect of the spiking control on the system state is to instead measure the work done on the system, by considering the kinetic energy before and after a neuron spikes. Specifically, kinetic energy is computed as $E^{\text{kinetic}} = \frac{1}{2}mv(t)^2$ (considering a unit mass, $m = 1$). Based upon the difference in kinetic energy before and after an instantaneous spike, the work done can be expressed as:

$$W = \sum_{i=1}^{N} \sum_{j=1}^{K_i} \lim_{\epsilon \to 0} \frac{1}{2} m(v(t_j^i + \epsilon)^2 - v(t_j^i - \epsilon)^2).$$

Effectively, this measures the change in kinetic energy before vs after the spike impacted the system. Notably, when a spike occurs from neuron i, the change in velocity is specifically b_i, such that

$$W = \sum_{i=1}^{N} \sum_{j=1}^{K_i} \lim_{\epsilon \to 0} \frac{1}{2} m((v(t_j^i - \epsilon) + b_i)^2 - v(t_j^i - \epsilon)^2).$$

In Fig B in S1 Appendix, we show measures of energy input in the three control paradigms (continuous control, filtered spiking control, and spiking control) for the example SMD control of Fig 7. We compare both the total work done on the system (Fig BA in S1 Appendix) and the total number of spikes in the two spiking methods (Fig BB in S1 Appendix). As shown, there is no universal energy advantage of our spiking algorithm from a control-theoretic perspective. Rather, our contribution lies primarily in the formulation of the spiking control scheme, in the sparsity of the resulting spiking activity, and in the interpretability of our model. Our paradigm offers solutions for neuroscience models where spikes need to be sparsely computing control signals (we included a simple example of this in Fig 9) or for any control applications where an impulsive control signal is needed.

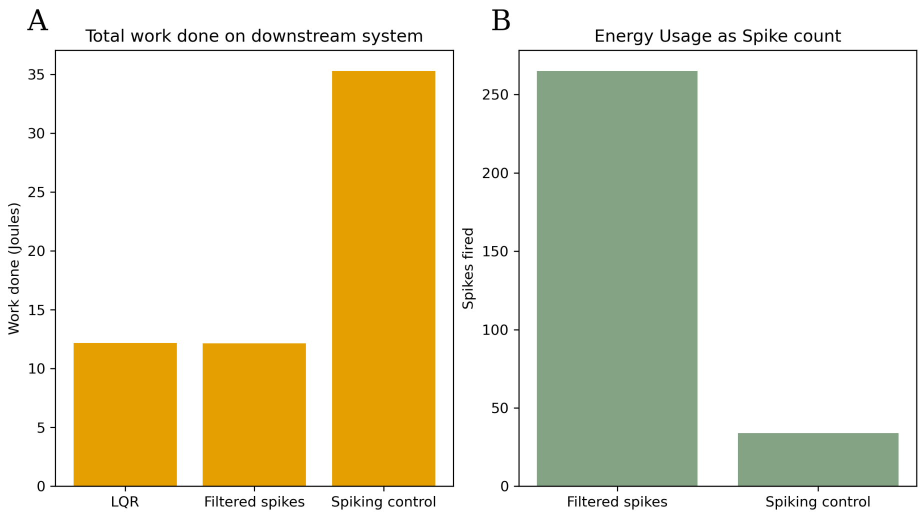

Figure B: Control input measures. A: Measures of work done in continuous, filtered spikes, and spiking control for a simple control task of a 2D linear SMD. B: Spike count for the same task for filtered spikes and spiking control.

Noise levels examples

In Fig 5 we showcase how the network is robust to the injection of noise in the neuron's voltages. We identified the source of this robustness in the local nature of the spiking rule, which refers to the fact that each neuron independently monitors a projection of the global prediction error (the distance between the plant's state and the target) within its own membrane potential. The same robustness property is showed in Fig 8 with the example of cell silencing and demonstrated by Boerlin et al., 2013. If noise causes an "erroneous" spike, that spike still provides a "kick" to the plant (or more generally for SCNs, a global effect on the network's dynamics), which is immediately reflected in the feedback signal $\mathbf{x}(t)$. Because the rule is local, every other neuron in the network is instantly affected by this change in the global error through its membrane potential (without needing direct connection to the noise-influenced neuron) and can spike accordingly to redirect the system towards the target. If, on the contrary, the noisy spike happened to move the system closer to the target, the other neurons simply "wait" longer to fire their next spike, as their local membrane potentials have been pushed further from their thresholds. This is true for the relatively contained level of voltage noise in Fig5. The effect of voltage noise can be increased by increasing our noise scaling factor, σ. As the injected noise values are drawn from a normal distribution, σ represents the standard deviation of such distribution. We will consider the same 4-neurons example of Fig 5, with different levels of noise. We identified three main network responses based on the noise levels. For $\sigma \leq 0.1$, the network spiking regime

and the control performance are both not heavily affected by the noise, as described above. This means the network retains its sparsity in spiking and manages to control the plant well. This is shown in the example of Fig 5, as well as in Fig CA in S1 Appendix. For values of $0.1 \leq \sigma \leq 1$, the network loses its sparse regime: the noise is driving a lot of neurons to spike. However, the control performance is still not heavily affected: all of the additional spikes produced are counter balanced by the network, which in turn outputs other spikes that kick the system in the opposite direction. This ping-pong effect pushes the network into an implausible spiking regime, but preserves the ability to trace the target (Fig CB in S1 Appendix). Finally, for $\sigma \geq 1$, the network spiking activity is extremely high and all sparsity is lost. Additionally, the network cannot successfully counter-spike to trace the target, as the neurons are constantly being pushed over their threshold by the high noise level. The control performance is affected, as shown in Fig CC in S1 Appendix. These same effects to voltage noise have been described for SNCs in general by the work of Calaim et al., 2022.

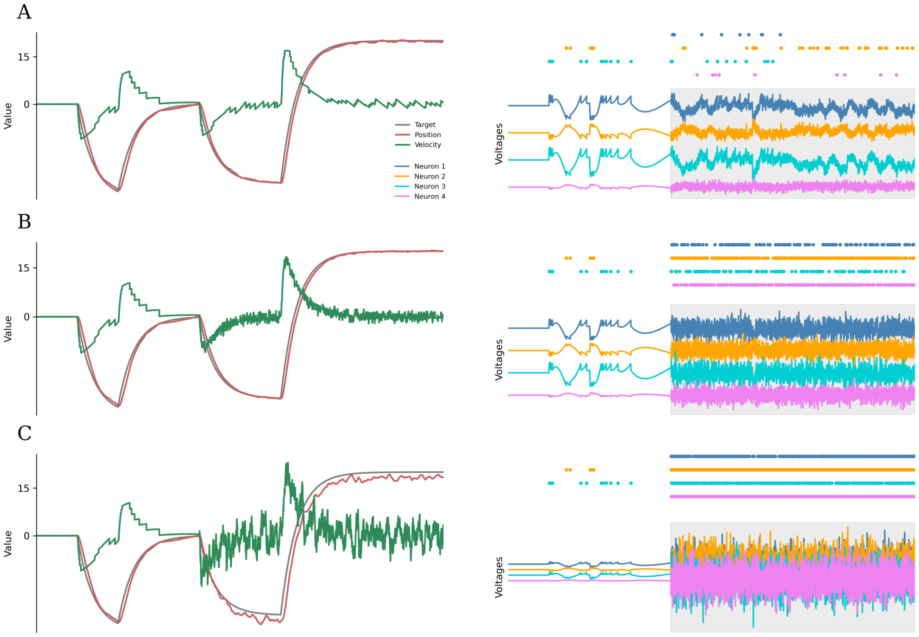

Figure C: SMD control with different noise levels. A: Simulation of an SMD control by a network of $N = 4$ neurons with a noise scaling of $\sigma = 0.08$. On the left: position, velocity, and target of the SMD. On the right: spikes and voltage traces for the four neurons. B: Identical simulation with a noise scaling of $\sigma = 0.2$. C: Identical simulation with a noise scaling of $\sigma = 2$.
