# Supplementary material for: Spiking neurons as predictive controllers of linear systems
Source: PLoS Comput Biol. 2026 Jul 9;22(7):e1014432. doi: 10.1371/journal.pcbi.1014432 (PMC13384403; doi:10.1371/journal.pcbi.1014432)
Supplement: Attachment — Submitted filename: Response to Reviewers.pdf [file pcbi.1014432.s003.pdf]

Response letter – Spiking neurons as predictive controllers of linear systems

Paolo Umberto Agliati

Feb 2026

We thank the reviewers for their careful reading of our manuscript and for the constructive and detailed comments. We have revised the manuscript accordingly, with the aim of strengthening the formal presentation of the paper and making some assumptions more clear, while improving the exposition of the results. We updated our continuous-time formulation and the practical interpretation of spike integration, and we clarified the time-horizon assumptions of the control problem. We also addressed the discussion on stability and asymptotic behavior, referencing recent control theoretical results (Eilers et al., 2025) that apply to our setting under stated assumptions. We revised several passages to avoid stating any implication of optimality, and improved the discussion comparing spiking and filtered control formulations. Finally, an S1 Appendix section was added to expand on certain relevant examples proposed by the reviewers. We addressed all specific comments regarding clarity, figures, equations, robustness, reproducibility, and implementation details, and changed the manuscript accordingly. Below we provide detailed responses. For clarity, reviewers' comments are reproduced in italic, followed by our responses. Note: line numbers in the responses refer to the new manuscript and not to the track changes version.

Reviewer 1

0.1 Conceptual points: Heuristic vs Formal theoretical framework

1. *The paper uses continuous time formulation of the problem but use a discrete time Dirac delta function. If the formalism is in continuous time, then delta(0) is infinity, not 1. If as it seems to be the case, the authors consider a delta that is zero everywhere and a mass of 1 at zero, then the developments must be expressed in discrete time.*

   We addressed this discrepancy in the revised manuscript (Materials and Methods section, line 765 onward) to avoid ambiguity between continuous-

and discrete-time representations. We still define spikes as Dirac delta distributions, thus maintaining the continuous time formulation. While the Dirac distribution itself is not evaluated pointwise, we now consider, in the derivation of the network, the integral over such spikes in a time interval (Δt). In practice, this is computed by the function $\Gamma(\mathbf{s})$ and corresponds to counting the spikes events in said interval, obtaining binary-valued spike variables (stored in the vector $\mathbf{o}$) while preserving a continuous-time formulation.

2. *The foregoing point is problematic because the loss function (Eqn. 12) is not well defined since the continuous-time version of Dirac delta is not in L2, so the norm of "s" is not defined (it is +infinity).*

   We agree with the reviewer that the L^2 norm of a Dirac distribution is not well defined. In the revised manuscript, we have reformulated the loss to consider a time-integrated spike signal rather than a norm the Dirac distribution itself. As mentioned in our response to question 1, instead of the term $||\mathbf{s}||^2$, the loss now contains a function $\Gamma(\mathbf{s})$ that takes the integral of the spike signal over a finite time window (Δt). This change is now made explicit in the definition of the loss function and propagated in the rest of the derivation (Materials and Methods, Derivation, line 782 onward) where the discrete spike vector $\mathbf{o}$ (which contains binary values instead of Dirac delta distributions) is distinguished from the continuous valued spike trains $\mathbf{s}$.

3. *There may be confusion about the time horizon: the parameter "f" is used as a time horizon but it was unclear whether the control problem was defined in finite horizon, or infinite horizon, or as receding horizon. This is potentially problematic as the derivation of the voltage dynamics is based on the assumption that the parameter "f" is fixed, which is not true in the finite horizon case. In fact in general, "Af" does depend on time through this parameter. It is possible that the approximation derived by ignoring this is not too bad but again, it is more a heuristic than a principled theory.*

   We have now clarified that the control problem is defined over an infinite horizon. The parameter f does not define a finite planning horizon, but rather a fixed temporal window over which instantaneous loss comparisons are computed. We now explicitly state this distinction and clarify that while certain system matrices depend on f, f itself is fixed and does not evolve with time. In Discussion, a paragraph ("Features of the control scheme", line 557) has been added to the manuscript to make these assumptions explicit.

4. *The paper is lacking formalism about the conditions under which optimality can be achieved. Currently the greedy spiking condition is derived such that the loss with a spike is lesser than the loss without spike, but this does not prove optimality. This is however clearly suggested in the text when the authors write that the spiking controller tracks the LQR control gains,*

which may be a particular case of their chosen simulation settings as optimality in general was not established. This is discussed in the paragraph on assumptions and constraints but it arrives late and other parts of the text suggest otherwise.

We emphasize that the manuscript does not claim optimality of the proposed control scheme. The greedy spiking condition is derived to ensure local loss reduction, not global optimality. We have revised the text to ensure that no statements suggest that the controller tracks or approximates an LQR solution. In particular, we clarified that references to LQR-tracking behavior (Section 0.2 Specific points - point number 4. in this review, lines 398 onward in the manuscript) pertain to the filtered spikes approach (where the network is indeed encoding an LQR signal, see Materials and Methods, Filtered spiking controller) and not to our main proposed algorithm. We also moved and expanded clarifications earlier in the manuscript (line 121) and explicitly referred readers to the assumptions and constraints section of the Discussion where this is addressed, as well as the Materials and Methods section, where the filtered spikes approach is derived.

5. *In the same vein, the article may currently overstate the performance of the approach by suggesting that it achieves control. What seems guaranteed is that each spike decreases the loss but less is known about the fact that the target can be reached. An argument about the asymptotic behaviour of the scheme is lacking. How do the cumulative effect of many spikes allow zeroing the error?*

   We thank the reviewer for raising this important point. We agree that the original manuscript did not provide a formal proof of asymptotic stability or convergence to zero error for our control paradigm. However, since sending our first manuscript, a recently published preprint (Eilers et al., 2025) took our work as a case study, and proved stability of our control over certain uniform bounds (see Lemma 12 of their work), which for us are defined by the spike strength mapping $\mathbf{B}$. We refer to this paper in the Discussion (in the new "Features of the control scheme" paragraph, line 557 onward) to show how our spiking algorithm provides stable control (albeit without guaranteeing an optimal solution).

6. *I believe that the comparison between the authors' approach and the filtering approach, where spike trains are transformed into filtered, rate-based approximation was artificial because in fact the filtering is clearly also performed here but by the biomechanics. Hence what the authors present as alternative views my collapse essentially to the same formalism dependent on which sub-part of the model filters the spike trains.*

   We agree that the distinction between spiking and filtered formulations can be partly artificial. The role of a filtering element in the paradigm depends on the modeling choice we operate and the hypothesis we consider for the specific research question. In the revised manuscript, we explicitly

acknowledge that the filtering effectively occurring in the physical system rather than within the network is indeed a modeling choice, although letting the computation of the control signals be instantaneous does indeed represent a new modeling paradigm for SNNs. We have revised the Results section (line 427 onward) to make this relationship explicit and to avoid overstating the conceptual separation between the two views. We included a biologically relevant example of our paradigm where the spikes are directly mapped to control the displacement of some fiber units (modeled as spring-mass-dampers) which in turn continuously affect the position of a point arm (In the Results, Fig.9). In this example, the various mapping elements are set up entirely based on the specific control task at hand. We do maintain that previous works employ filtering as a conversion method for the control signal: the filter converts discrete spike events into continuous filtered spike traces. In our original SMD example, the filtering is simply scaling and orienting the spikes in state space, but it is not altering the resulting control input (which remains impulsive rather than continuous in nature).

0.2 Specific points

1. *On and around line 242: perhaps the long discussion about the relationship between position and velocity as a constraint is superfluous for the readership of PLoS CB.*

   The discussion has been shortened and redundant sentences removed, as suggested.

2. *Legend of Fig. 4: I was missing the argument about why the network never spikes in the reactive case.*

   We clarified in the main text and expanded the Figure 4 legend to explain why the network does not spike in the reactive case.

3. *Line 401: There is an example of statement suggesting that the controller approximates LQR which strongly but misleadingly suggests that the spiking controller is optimal.*

   As noted above, we clarified that the paragraph in question refers to the filtered spikes approach and not to our direct spiking method, and we explicitly state that our algorithm does not approximate an optimal control solution.

4. *The robustness analysis is extremely interesting and important.*

   We expanded the discussion of robustness (line 460 onward) and explicitly related it to the local, greedy spiking rule, referencing prior work (Boerlin et al., 2013) that establishes robustness properties under neuron silencing and perturbations.

5. *Eqn. 21: make mu divided by 2.*

   The factor of 1/2 has been corrected, and the equation now reflects the updated definition used in the simulations.

6. *Line 719: Example of a statement that the network is able to perform control but what is shown at this stage is that the network decreases the loss locally, which is not the same as solving the control problem.*

   We did not encounter the statement in this specific line, however, we revised the manuscript to reference the stability proof of Eilers et al., 2015, and we openly state the lack of a formal stability analysis in our work.

Reviewer 2

0.3 Conceptual points: spiking bio-plausibility and discussion

1. *The assumption that only one neuron fires at any one time is implausible, especially since real action potentials are extended in time. While the manuscript hints at possible solutions in the paragraph starting at line 142, it would make the presentation much stronger if a working solution was presented, e.g. the threshold scaling that is mentioned in line 154. I would like to see such a demonstration in a revised manuscript.*

   We thank the reviewer for the insightful comment. We agree that the assumption of strictly asynchronous spiking is biologically implausible. As the paper is seen as a proof of concept, we initially took the approach that simplified the most the explanation and illustration of our paradigm. The asynchronous firing rule is conceptually closer to the mathematical derivation of our network, which develops based on a single neuron perspective. However, we agree that a working solution beyond this idealized regime substantially strengthens the framework. Lifting the asynchronous firing constraint raises issues relative to the fully local property of the network. For instance, in a system where the signal reaches the neurons with a time delay, multiple neurons would independently reach their threshold and fire, inputting unnecessary control signals and compromising the sparsity of the code. Important to note that for simulations with zero delays that use very small timesteps, the asynchronous firing regime is naturally maintained, but for larger timesteps or in the presence of delays this is no longer feasible. We generalized our derivation of the network to render it functional in case of simultaneous spiking, and in the case in which the cost reduction reaches the neurons with a time delay (see Materials and Methods, line 765 onward). The solution indeed involves a spike-threshold adaptation mechanism, where the threshold of each neuron is scaled by the neuron's past activity, through a parameter α. We also include additional results in the S1 Appendix showing the effect of delays on network

behavior, in the presence or absence of our spike-threshold adaptation (Supplementary Fig.1 in S1 Appendix).

2. *The real advantage of the proposed spiking control scheme vs. filtering approaches does not become very clear in the current manuscript. Is it the total energy expended on spiking? In that vein, why is "Energy Usage calculated as effected acceleration onto the system in each simulation" (line 329) and not as work done on the system? Why are spike costs not included into the energy usage computation? Could the authors please plot and discuss that? There seems to be a supplemental figure produced by the figure 7 notebook, which shows exactly that – it should be included in the main paper.*

   We have revised the discussion comparing spiking and filtering approaches. We have now included additional plots in the S1 Appendix (Supplementary Fig.2 in S1 Appendix) that account for spike-related costs and measure the work done on the system in both control approaches. The Discussion (line 437 onward) and the S1 Appendix now makes clear that there is no universal energy advantage from a control-theoretic perspective, but the paradigm is useful in control problems where the inputs need to be sparse and direct. Models with bio-plausible constraint often need to include such limitations. Thus, our contribution lies primarily in the formulation and interpretation of the spiking control paradigm.

3. *A really important feature of biological motor control is signal-dependent noise, which leads to minimally intervening controllers. While the spiking cost μ in eqn 3 promotes small interventions, the system is currently modeled as noise-free. Could the authors please comment on (or even demonstrate) the effects of noisy spiking?.*

   We introduced noise in the voltages and demonstrated the robustness of the control to noisy voltage fluctuations in the Results (see Fig5). We added a discussion regarding how noise can be introduced at the voltage level. We relate this to prior probabilistic spiking models that guarantee robustness, and we show in Results (line 284 onward) how our control performance is not heavily affected by noisy voltages. In our paper, we did not examine signal dependent noise and its complex effects on the resulting control, as we deemed such explorations out of scope for this work. However, our proposed framework can accommodate for similar extensions, potentially in a follow up paper where other interesting control features can be studies (nonlinear plants, meta-parameter discounting, etc).

4. *While the 10 SMDs are a natural extension of the minimal example, the approach would be advertised much better by a standard example of articulated motor control, such as balance model or a two-link arm executing a reaching movement. I would like to encourage the authors to add one such example to the manuscript.*

We agree that articulated control is an important direction, but believe that a full nonlinear arm model is beyond the scope of the present work. However, we showcased a relevant extension including the bio-inspired control of a linear point arm model (in the Results, line 475 onward). The spiking control is actuated by the network on multiple SMDs, used here as models of fiber units (the use of oscillatory dynamics to model fiber displacement is present in the literature). The state of each unit then affects the position of the point arm, which through our algorithm is able to follow a target trajectory. Since single spikes cause oscillations in the SMDs, which in turn continuously affect the state of the point arm, the network maintains a very sparse spiking activity. The scheme of the example, the tracing of the trajectories, and the relevant activities are showed in Fig.9 of the manuscript.

0.4 Specific points

1. *The figure resolution is far too low. Especially in figure 4, I can only guess which lines I'm supposed to see. Please provide higher resolution figures. But it really helps to have the jupyter notebooks.*

   All figures have been regenerated at higher resolution to meet the journal's requirements. Note that the figure in the review document are at a lower resolution than the one included as separate files in the submission.

2. *I was able to run all notebooks, but the notebooks for fig 6 and 8 produced empty plots only?*

   We verified the notebooks on multiple systems and corrected minor configuration issues to ensure that all figures render correctly.

3. *In the derivation of the threshold and the membrane voltage, I don't understand how μ gets in the numerator of eqn. 22 – should there be a factor 2? Likewise in eqn. 5. This definition is also used in line 121 of* `lib_sim.py`?

   These equations have been corrected, and the updated definitions are now consistent throughout the manuscript and the code.

4. *cairosvg seems to be a dependence of the notebooks, too. Please include into the README.*

   The dependency has been added to the README file.
