# Supplementary material for: Spiking neurons as predictive controllers of linear systems
Source: PLoS Comput Biol. 2026 Jul 9;22(7):e1014432. doi: 10.1371/journal.pcbi.1014432 (PMC13384403; doi:10.1371/journal.pcbi.1014432)
Supplement: Attachment — Submitted filename: Response_to_reviewers.pdf [file pcbi.1014432.s004.pdf]

# Response letter – Spiking neurons as predictive controllers of linear systems

Paolo Umberto Agliati

Jun 2026

We thank the reviewers for the detailed comments and feedback. We have revised the manuscript accordingly, changing some terminology to prevent ambiguities. We improved the nomenclature and clarity in the code as well, for reproducibility. We addressed and clarified some theoretical points in this letter, and included new simulations to further exemplify these points both in the main manuscript and in the S1 Appendix. These further analyses include an assessment of robustness to noise in the network's voltages, and the response to the network to perturbations in the arm reaching task of Fig. 9. Below we provide detailed responses. For clarity, reviewers' comments are reproduced in italic, followed by our responses.

## Reviewer 1

1. *The authors have addressed my comments. There remain a point that I failed to fully understand about the fact that in the reactive case the network does not spike (Fig. 4). Apologies if it is simple but I was worried that the notions of predictive control and reactive control were misleadingly opposed. Take the stochastic optimal control formalism, it is purely reactive in the sense that it is a state feedback controller in the linear case, whereas there is some predictive scheduling of the feedback gain matrix. Could the author consider reformulate the explanation? Is it that what is called "reactive case" corresponds to f=0? In which case there is a risk that reactive be misunderstood for "feedback", while the latter can exhibit some predictive structure.*

   We value the input from the reviewer highlighting the potential ambiguous use of the terms reactive and predictive in our model. We will first explain the lack of spiking for the reactive case. We will then trace some parallels between what can be considered "reactive" and "predictive" in the stochastic optimal control formalisms, and how that maps onto our approach. This will hopefully then explain why we ended up adopting the two terms in the manuscript.

**The reactive network is silent when physically constrained** The failure to spike in the physically constrained SMD example is a direct consequence of the reactive control case, in which indeed $f = 0$. In a physically constrained SMD, the target variable is the position, but the control signal (the spike) can only directly influence the velocity. Because an impulse cannot "teleport" the mass, a change in velocity does not instantaneously change the mass's position. In state space, a spike on the velocity will at first drive the trajectory away from the target (at least for those initial conditions of the SMD). Consequently, at $f = 0$, the loss (which measures the distance between the current position and the target) is increased after the spike (see Fig. 3A). Since the greedy spiking rule dictates that a neuron only fires if $L_i^{\mathrm{s}} \leq L^{\mathrm{ns}}$, the threshold is never reached, and the network remains silent.

**Reactiveness and predictiveness in optimal control formalisms** In stochastic optimal control formalisms (like the Linear Quadratic Regulator (LQR) or Linear-Quadratic-Gaussian (LQG)), an element that can be described as "reactive" is present during the control execution, because a control signal $u$ is immediately (and continuously) generated based on the current state feedback $\mathbf{x}$. This is related to the closed loop feedback nature of the control, since as long as $\mathbf{x}$ is available to the controller, a signal $u$ will be computed. The "predictive" nature of these approaches is to be found in the gain matrix $\mathbf{K}$ which is "predictively" calculated by solving an algebraic Riccati equation that minimizes a cost function over an infinite or finite future horizon. These notions of "reactive" and "predictive" features of the control do not map directly to reactiveness and predictiveness as described in our approach. I will indicate "reactive" and "predictive" with quotes when referring to the stochastic optimal control formalisms, I will leave them without quotes when referring to our approach.

**Link to our approach and name choice** Our spiking control, in both the reactive and predictive cases (as they are described in the manuscript) is operating in a closed loop feedback regime every time the network spikes. The system evolves in open loop until a spike is produced. The spike event, when mapped onto the system, closes the loop and causes an instantaneous switch to a feedback control. After the spike has affected the system, it will evolve in open loop until the next spike. This means that both our predictive and reactive cases are operating with the "reactive" element that we described above for stochastic optimal control formalisms, which is what guarantees that feedback control is present. Our distinction between predictive and reactive is thus only based on the length of the future time window $f$ for our infinite horizon control problem. As mentioned, the "predictiveness" in classic optimal control approaches is given by computing the gain matrix $\mathbf{K}$. In our case, such "prediction" is also pre-computed

and then evaluated at every simulation timestep. Each neuron computes whether an instantaneous "kick" (spike) will improve the system's state $f$ seconds into the future. To do so, the neurons have access to the loss (distance to the target), which includes a measure of the future state of the system $\mathbf{A}_f = e^{\mathbf{A}f}$. This predictive element is present in all approaches, the only difference being that in the reactive case $f = 0$, which means the loss is evaluated as the distance from the target immediately after the spike is produced (see Fig.3). One could argue that a more indicative name for this approach would then be "immediate" or "instantaneous" spiking control. While in principle this is true, this name could be seen as even more ambiguous, given the fact that we are operating with instantaneous kicks (spikes) in our impulsive control paradigm. This could lead the reader to think that only the reactive (now called instantaneous or immediate) spiking regime actually operates with impulsive control, which is of course not the case. To avoid this confusion, we decided to stick with the reactive name instead.

# Reviewer 2

1. *eqn 5, also line 812 : $\mathbf{r}_i$ is a vector (spike train of neuron i), but the rest of the equation is scalar? Please explain.*

   Throughout the text, bold characters are vectors and matrices (vectors are lowercase and matrices uppercase), while non-bold characters are scalars. In Equation 5, the meta-parameters $\mu$ and $\alpha$ are acting as a spiking cost and a threshold scaling, respectively. The resulting value of the threshold for a single neuron $i$ ($T_i$) is also a scalar. While the other entries of the equation ($\mathbf{r}_i$, $\mathbf{b}_i$, and $\mathbf{A}_f$) are vector or matrices, which is why they are inserted in bold. NOTE: all subscripts and superscripts are also not bold.

2. *line 267: able to observe future states – predict future states*

   Corrected

3. *line 312: "The local nature of the spiking rule helps with preventing this noisy spiking to affect the resulting control performance." How do you know it's the local nature? Also, could you please specify the noise you injected and the limits where performance breaks down?*

   The "local nature" of the spiking rule refers to the fact that each neuron independently monitors a projection of the global prediction error (the distance between the plant's state and the target) within its own membrane potential (we now mention this explicitly in line 300, and refer to the derivation). This is the only candidate property able to guarantee robustness in the framework. Each neuron encodes this prediction independently, and has no access to the state of other neurons (see line 554 onward). We will briefly explain how the original greedy spiking rule pro-

vides robustness to the network, and describe in which way the effects of such rule are applicable to our paradigm.

**Greedy spiking rule and robustness** The work of Boerlin et al., 2013 (from which we adopted our spiking rule) demonstrates that while individual neurons may appear to fire stochastically or indeed respond to noise, the population as a whole acts as a "deterministic super-unit". If noise causes an "erroneous" spike, that spike still provides a "kick" to the plant (or in the case of Boerlin et al. 2013, a global effect on the network's dynamics), which is immediately reflected in the feedback signal $\mathbf{x}(t)$. Because the rule is local, every other neuron in the network instantly sees this change in the global error through its membrane potential (without needing direct connection to the noise-influenced neuron) and can spike accordingly to redirect the system towards the target. If, on the contrary, the noisy spike happened to move the system closer to the target, the other neurons simply "wait" longer to fire their next spike, as their local membrane potentials have been pushed further from their thresholds. Noise on the voltage can be seen here as a source of alteration of the spiking patterns of the neurons. An extreme example of the same concept can be shown by cell silencing, where neurons are prevented from spiking from a certain time until the end of the simulation. In their inactivation demonstration (Fig. 7G), Boerlin et al. 2013, show that suddenly silencing half the neurons has "essentially no effect on the network performance" (we show a similar effect in Fig. 8 of our manuscript).

**Robustness in our work** The same local logic applies for noise robustness: each neuron in the network "detects" the altered error and adjusts its activity accordingly, maintaining a stable trajectory. Our approach does not provide any additional source of robustness in the control outside of the spiking rule (and thus of the network structure) derived by Boerlin et al., 2013. In fact, our work can be seen as an extension of the same principle in Boerlin et al. 2013, applied to controlling downstream systems. This was shown in the Materials and Methods section, and is now also stated more clearly in the Results section (Spike-based control paragraph, line 149 of the manuscript). Effects of robustness to voltage noise on SCNs specifically are also illustrated through a geometric perspective in the work of Calaim et al., 2022, where the noise causes at first loss of sparsity in the network's spiking, and for higher levels compromises the network's performance. To inject the noise in the example of Fig. 5 of our work, we initialize different random values drawn from a normal distribution with a standard deviation $\sigma = 0.08$. We then simply add the resulting values directly to the voltages of each neuron. In the S1 Appendix, we identified three effects of progressive noise levels on the network. We illustrate three simulations controlling an SMD (identical to the one in Fig. 5) with three different levels of noise. The first regime is

a level of noise the network is robust to ($\sigma = 0.08$, as in Fig. 5). Spiking activity starts losing sparsity in the second example, at around $\sigma = 0.2$, but in this regime the control performance is still mainly unaffected. This happens because although the network is now spiking excessively, neurons are still able to successfully compensate the effect of the noisy spiking by simply counter-spiking (kicking the velocity in the opposite direction). The control then fails to trace the target accurately for very high sigma values, at around $\sigma = 2$.

4. *Fig. 6: "As described in S1 Appendix, the measure of acceleration is chosen for simplicity of interpretation in our framework. However, measures of work done on the plant can also be adopted to describe the energy input" What is the "simplicity of interpretation"? I propose to call this quantity 'integrated acceleration' instead*

   The measure of effected (integrated) acceleration can be described as the summation of all mapped spike inputs into the system. The quantity can be defined by:

   $$W = \sum_{i=1}^{N} \sum_{j=1}^{K_i} \int_0^{T_{\text{end}}} b_i \delta(t - t_j^i)\, \mathrm{d}t\,,$$

   as described in the S1 Appendix. Such sum would, more practically, coincide with **Bs** (see line 378 and Fig.6 of the manuscript). This allows us to interpret the work done by the system in terms of matrices that are directly present in the network derivation, and that describe clearly the amount of acceleration that is injected into the system (by mapping the spikes onto the plant) during the simulation. The measure of work done is calculated as follows:

   $$W = \sum_{i=1}^{N} \sum_{j=1}^{K_i} \lim_{\epsilon \to 0} \frac{1}{2} m(v(t_j^i + \epsilon)^2 - v(t_j^i - \epsilon)^2)$$

   (as in S1 Appendix). Effectively, this measures the change in kinetic energy right before vs right after the spike impacted the system. The quantity forces us to introduce a unitary mass $m$ that is never used in the derivations or demonstrations in the paper, and to also consider an infinitely small window $\epsilon$ that is applied to the spike time (in order to evaluate the change in velocity before and after the kick is produced). Since the spike time was already defined with its own time window, we thought this could be more convoluted then simply considering the effect of each mapped spike onto the system. We agree integrated acceleration is a more representative name, we updated the manuscript accordingly.

5. *line 354 ff: for the predictive control, you need a state predictor. What system is doing the predicting? Is that spiking too? Please comment.*

   The prediction is pre-computed and made available to the neurons in each timestep as the losses in the spike and non-spike conditions are compared

(to compute the spike decision). The future state of the linear system is pre-computed based on a fixed time window $f$ as the matrix exponential of the state matrix $\mathbf{A}$ (Eq.4, line 131):

$$\mathbf{A}_f = e^{\mathbf{A}f}$$

.

$\mathbf{A}_f$ is a fixed quantity for the given control task and is encoded in the network weights. This way, the spiking neurons (their voltages and thresholds) include $\mathbf{A}_f$ as one of the measures that determines each spiking decision, and are in fact operating the prediction within the control paradigm. Such measure is evaluated both starting from the current system state, $\mathbf{A}_f\mathbf{x}_0$ and starting from the end point of a mapped spike, $\mathbf{A}_f(\mathbf{x}_0 + \mathbf{b}_i)$ (see Eq. 10 and 11 on the manuscript and the relative derivations).

6. *line 388: "With an adequate future time window f" what is that? Can it be related to the dynamical parameters of the system (spring constant vs. dampening)? If this approach is going to be useful, knowing how to choose f is important. Please explain*

   We now explicitly mention in the Varying meta-parameters and Assumption and Constraint paragraphs (lines 365 and 566), that the choice of $f$ is currently tuned manually for each specific use case. The manuscript explicitly categorizes the predictive window as a meta-parameter that must be adjusted to fit the scale and dynamics of the system being controlled. In most other stochastic optimal control paradigms, meta-parameters also need to be adjusted to specific use cases. In Fig. 6, we show how changing this meta-parameter (in conjunction with changing the spike cost $\mu$) can substantially affect the control performance. Of course, varying values of $f$ until a good control performance is reached is not an actionable strategy when facing the control of a real system. This manuscript is oriented towards demonstrating that control operated by a spiking neural network derived from a greedy spiking rule is possible. In a second manuscript, we are currently working on controlling systems with unknown dynamics, which requires the network to learn (or at least have some form of discount on) its meta-parameters, included $f$. In this cases, $f$ can be randomly initialized (or set to a very small or very large number) and then its value can be discounted based on the knowledge acquired on the system, until the value of $f$ that guarantees the best control performance is reached. This way, $f$ would indeed end up being somehow related to the dynamical variables of the plant (in our SMD case, spring constant and dampening), and different control problems would converge to different values of $f$.

7. *line 483 ff, arm reaching task. What were the parameters used here? Is this predictive control? How long was the f window? How does the arm respond to unexpected perturbations on the way to the target?*

For the arm reaching task, predictive control is used with a future time window $f$ of 2 seconds (line 720). We have now included all the parameters of all simulations (including the one in Fig.9) in the "Simulation and network setup" section, under Materials and Methods. As for the coupled oscillator and any other linear system, the control in the arm reaching task is robust to unexpected perturbations to the trajectory. In Fig. 9B we included an example trajectory where the velocity of the arm is suddenly changed in two different directions on its way to the target. After the perturbations, the network is able to redirect the trajectory and trace the target. Perturbing the position of the arm gives similar results (but we did not include it as it would not be physically feasible).

## 0.1 Specific points

1. *README: 'control' is also a required library.*

   Library was added to the README.

2. *fig 6 is still empty for me (simulations run, but plot shows nothing)*

   We found an issue in some systems where (due to the Matplotlib backend) some data was not kept in subsequent cells, and the plots displayed empty. We adjusted to have all the simulation and plotting in the same cell, so the code outputs the plots. We tested it online with Google Colab and in various systems, it should now be completely reproducible.

3. *in fig 9: plt.savefig('figs/fig11/axBfig11.svg', dpi=300) should this be renamed to fig9?*

   Corrected to 'axBfig9'. (NOTE: for layout issues I plot the axs (subplots) of fig9 individually, that's why this is only the axB of the figure).

4. *in fig 10: line 26 Bk = np.zeros((2*M, N)) should this be Nk? plt.savefig('figs/fig9.png', dpi=300) should this be fig10?.*

   Corrected to Nk, corrected to fig10.png.

5. *in fig 7,11: figure 11 is saved as figure 10.*

   Corrected all the name files for the plots.
