# Supplementary material for: Spiking neurons as predictive controllers of linear systems
Source: PLoS Comput Biol. 2026 Jul 9;22(7):e1014432. doi: 10.1371/journal.pcbi.1014432 (PMC13384403; doi:10.1371/journal.pcbi.1014432)
Supplement: Peer review history

# Peer review: Decision Letter 0

**DOI:** 10.1371/journal.pcbi.1014432.r001

**Authors:** Gunnar Blohm, Marieke van Vugt

11 Dec 2025

PCOMPBIOL-D-25-02142

Spiking neurons as predictive controllers of linear systems

PLOS Computational Biology

Dear Dr. Agliati,

Thank you for submitting your manuscript to PLOS Computational Biology. After careful consideration, we feel that it has merit but does not fully meet PLOS Computational Biology’s publication criteria as it currently stands. Therefore, we invite you to submit a revised version of the manuscript that addresses the points raised during the review process.

Please submit your revised manuscript by Feb 10 2026 11:59PM. If you will need more time than this to complete your revisions, please reply to this message or contact the journal office at ploscompbiol@plos.org. When you’re ready to submit your revision, log on to https://www.editorialmanager.com/pcompbiol/ and select the 'Submissions Needing Revision' folder to locate your manuscript file.

Please include the following items when submitting your revised manuscript:

* A rebuttal letter that responds to each point raised by the editor and reviewer(s). You should upload this letter as a separate file labeled 'Response to Reviewers'. This file does not need to include responses to formatting updates and technical items listed in the 'Journal Requirements' section below.

* A marked-up copy of your manuscript that highlights changes made to the original version. You should upload this as a separate file labeled 'Revised Manuscript with Track Changes'.

* An unmarked version of your revised paper without tracked changes. You should upload this as a separate file labeled 'Manuscript'.

If you would like to make changes to your financial disclosure, competing interests statement, or data availability statement, please make these updates within the submission form at the time of resubmission. Guidelines for resubmitting your figure files are available below the reviewer comments at the end of this letter

We look forward to receiving your revised manuscript.

Kind regards,

Gunnar Blohm, Ph.D.

Academic Editor

PLOS Computational Biology

Marieke van Vugt

Section Editor

PLOS Computational Biology

**Journal Requirements:**

1) Please ensure that the CRediT author contributions listed for every co-author are completed accurately and in full.

At this stage, the following Authors/Authors require contributions: Paolo Umberto Agliati, André Rodrigues Urbano, Pablo Lanillos, Nasir Ahmad, Marcel van Gerven, and Sander Keemink. Please ensure that the full contributions of each author are acknowledged in the "Add/Edit/Remove Authors" section of our submission form.

The list of CRediT author contributions may be found here: https://journals.plos.org/ploscompbiol/s/authorship#loc-author-contributions

2) We ask that a manuscript source file is provided at Revision. Please upload your manuscript file as a .doc, .docx, .rtf or .tex. If you are providing a .tex file, please upload it under the item type u2018LaTeX Source Fileu2019 and leave your .pdf version as the item type u2018Manuscriptu2019.

3) Please amend your detailed Financial Disclosure statement. This is published with the article. It must therefore be completed in full sentences and contain the exact wording you wish to be published.

- State the initials, alongside each funding source, of each author to receive each grant. For example: "This work was supported by the National Institutes of Health (####### to AM; ###### to CJ) and the National Science Foundation (###### to AM)."

- State what role the funders took in the study. If the funders had no role in your study, please state: "The funders had no role in study design, data collection and analysis, decision to publish, or preparation of the manuscript.".

If you did not receive any funding for this study, please simply state: u201cThe authors received no specific funding for this work.u201d

**Reviewers' comments:**

Reviewer's Responses to Questions

**Comments to the Authors:**

**Please note here if the review is uploaded as an attachment.**

Reviewer #1: The paper provides a much needed development about control design with a spiking network connected to a mechanical system. The application to neuroscience research is clearly motivated by the need to build models of biological neural networks that achieve control to understand neural population activity and potentially develop model-based brain-computer interface. The topic is very stimulating, timely, the paper goes in the right direction and it seems highly relevant to the readership of PLoS Computational Biology.

My enthusiasm was tempered by several problems related to the mathematical grounds, as explained below. Before going into detail, it is important to emphasise why this is important: in order to develop a theory of control design with a spiking network, I believe that the mathematical foundations must be rock solid, which does not seem to be the case at present. However, the authors demonstrate through simulations that the developments do work, so perhaps it is sufficient to present the formalism as a heuristic approach that achieves control in these cases, rather than as a formal theoretical framework. If, on the other hand, the authors wish to establish a formal theoretical framework for control, I believe that the following points need to be addressed, but I understand if they are considered beyond the scope of the article or the journal:

1- The paper uses continuous time formulation of the problem but use a discrete time Dirac delta function. If the formalism is in continuous time, then delta(0) is infinity, not 1. If as it seems to be the case, the authors consider a delta that is zero everywhere and a mass of 1 at zero, then the developments must be expressed in discrete time.

2- The foregoing point is problematic because the loss function (Eqn. 12) is not well defined since the continuous-time version of Dirac delta is not in L2, so the norm of “s” is not defined (it is +infinity).

3- There may be confusion about the time horizon: the parameter “f” is used as a time horizon but it was unclear whether the control problem was defined in finite horizon, or infinite horizon, or as receding horizon. This is potentially problematic as the derivation of the voltage dynamics is based on the assumption that the parameter “f” is fixed, which is not true in the finite horizon case. In fact in general, “Af” does depend on time through this parameter. It is possible that the approximation derived by ignoring this is not too bad but again, it is more a heuristic than a principled theory.

4- The paper is lacking formalism about the conditions under which optimality can be achieved. Currently the greedy spiking condition is derived such that the loss with a spike is lesser than the loss without spike, but this does prove optimality. This is however clearly suggested in the text when the authors write that the spiking controller tracks the LQR control gains, which may be a particular case of their chosen simulation settings as optimality in general was not established. This is discussed in the paragraph on assumptions and constraints but it arrives late and other parts of the text suggest otherwise.

5- In the same vein, the article may currently overstate the performance of the approach by suggesting that it achieves control. What seems guaranteed is that each spike decreases the loss but less is known about the fact that the target can be reached. An argument about the asymptotic behaviour of the scheme is lacking. How do the cumulative effect of many spikes allow zeroing the error?

6- I believe that the comparison between the authors’ approach and the filtering approach, where spike trains are transformed into filtered, rate-based approximation was artificial because in fact the filtering is clearly also performed here but by the biomechanics. Hence what the authors present as alternative views my collapse essentially to the same formalism dependent on which sub-part of the model filters the spike trains.

Specific comments:

On and around line 242: perhaps the long discussion about the relationship between position and velocity as a constraint is superfluous for the readership of PLoS CB

Legend of Fig. 4: I was missing the argument about why the network never spikes in the reactive case.

Line 401: There is an example of statement suggesting that the controller approximates LQR which strongly but misleadingly suggests that the spiking controller is optimal.

The robustness analysis is extremely interesting and important.

Eqn. 21: Shouldn’t mu be divided by 2 as well?

Line 719: Example of a statement that the network is able to perform control but what is shown at this stage is that the network decreases the loss locally, which is not the same as solving the control problem.

Reviewer #2: The manuscript describes an approach for controlling linear systems directly with quasi-discrete control signals that are intended to mimic neuronal action potentials. This is an interesting idea, given that most previous work used either continuous controls or approximations thereof (smoothed spiketrains).

Spikes are modelled as point events in continuous time, the effect onto the system to be controlled is exerted by replacing each spike with a Dirac delta distribution. This approximation is common in theoretical neuroscience due to the stereotypicality of spikes, even though a real action potential has a temporal extension of several milliseconds. With this point event approximation, it is possible to derive the neuronal network parameters from the control objective, as long as the controlled system is linear. To illustrate the approach, a simple spring-mass-damper (SMD) system is controlled reactively and predictively. Spikes are generated if they lead to a reduction of a cost function comprised of a control cost and a spiking cost. For this neuron-wise descent to work, it is necessary that only one neuron fires at any one time, and that the effect of the cost reduction is instantly made available to all other neurons in the network. Especially the first assumption is somewhat implausible from a biological perspective. For the SMD system, it is then demonstrated that the spiking control works reactively and predictively with the right parameter tuning. The proposed approach is compared to two previously described control approaches: continuous LQR and spike-filtering for the approximation of LQR. Some differences are highlighted, but the discussion falls short of explaining the advantages of spike-based control vs. spike-filtered control. The approach is also applied to controlling SMDs connected in series with a larger number of neurons. It is demonstrated that this works too, even if a part of the neuronal population drops out. It is very helpful to have the jupyter notebooks in a git repo to reproduce the simulations and figures.

Major remarks:

- the assumption that only one neuron fires at any one time is implausible, especially since real action potentials are extended in time. While the manuscript hints at possible solutions in the paragraph starting at line 142, it would make the presentation much stronger if a working solution was presented, e.g. the threshold scaling that is mentioned in line 154. I would like to see such a demonstration in a revised manuscript.

- The real advantage of the proposed spiking control scheme vs. filtering approaches does not become very clear in the current manuscript. Is it the total energy expended on spiking? In that vein, why is "Energy Usage calculated as effected acceleration onto the system in each simulation" (line 329) and not as work done on the system? Why are spike costs not included into the energy usage computation? Could the authors please plot and discuss that? There seems to be a supplemental figure produced by the figure 7 notebook, which shows exactly that -- it should be included in the main paper.

- A really important feature of biological motor control is signal-dependent noise, which leads to minimally intervening controllers. While the spiking cost \mu in eqn 3 promotes small interventions, the system is currently modelled as noise-free. Could the authors please comment on (or even demonstrate) the effects of noisy spiking?

- While the 10 SMDs are a natural extension of the minimal example, the approach would be advertised much better by a standard example of articulated motor control, such as balance model or a two-link arm executing a reaching movement. I would like to encourage the authors to add one such example to the manuscript.

Minor:

- the figure resolution is far too low. Especially in figure 4, I can only guess which lines I'm supposed to see. Please provide higher resolution figures. But it really helps to have the jupyter notebooks.

- I was able to run all notebooks, but the notebooks for fig 6 and 8 produced empty plots only?

- in the derivation of the threshold and the membrane voltage, I don't understand how \mu gets in the numerator of eqn. 22 -- should there be a factor 2? Likewise in eqn. 5. This definition is also used in line 121 of lib_sim.py ?

- cairosvg seems to be a dependence of the notebooks, too. Please include into the README

**********

**Have the authors made all data and (if applicable) computational code underlying the findings in their manuscript fully available?**

The PLOS Data policy requires authors to make all data and code underlying the findings described in their manuscript fully available without restriction, with rare exception (please refer to the Data Availability Statement in the manuscript PDF file). The data and code should be provided as part of the manuscript or its supporting information, or deposited to a public repository. For example, in addition to summary statistics, the data points behind means, medians and variance measures should be available. If there are restrictions on publicly sharing data or code —e.g. participant privacy or use of data from a third party—those must be specified.

Reviewer #1: Yes

Reviewer #2: Yes

**********

PLOS authors have the option to publish the peer review history of their article (what does this mean?). If published, this will include your full peer review and any attached files.

If you choose “no”, your identity will remain anonymous but your review may still be made public.

**Do you want your identity to be public for this peer review?**  For information about this choice, including consent withdrawal, please see our Privacy Policy.

Reviewer #1: No

Reviewer #2: **Yes:** Dominik Endres, PhD

[NOTE: If reviewer comments were submitted as an attachment file, they will be attached to this email and accessible via the submission site. Please log into your account, locate the manuscript record, and check for the action link "View Attachments". If this link does not appear, there are no attachment files.]

**Figure resubmission:**

While revising your submission, we strongly recommend that you use PLOS’s NAAS tool (https://ngplosjournals.pagemajik.ai/artanalysis) to test your figure files. NAAS can convert your figure files to the TIFF file type and meet basic requirements (such as print size, resolution), or provide you with a report on issues that do not meet our requirements and that NAAS cannot fix.

After uploading your figures to PLOS’s NAAS tool - https://ngplosjournals.pagemajik.ai/artanalysis, NAAS will process the files provided and display the results in the "Uploaded Files" section of the page as the processing is complete. If the uploaded figures meet our requirements (or NAAS is able to fix the files to meet our requirements), the figure will be marked as "fixed" above. If NAAS is unable to fix the files, a red "failed" label will appear above. When NAAS has confirmed that the figure files meet our requirements, please download the file via the download option, and include these NAAS processed figure files when submitting your revised manuscript.

**Reproducibility:**

To enhance the reproducibility of your results, we recommend that authors of applicable studies deposit laboratory protocols in protocols.io, where a protocol can be assigned its own identifier (DOI) such that it can be cited independently in the future. Additionally, PLOS ONE offers an option to publish peer-reviewed clinical study protocols. Read more information on sharing protocols at https://plos.org/protocols?utm_medium=editorial-email&utm_source=authorletters&utm_campaign=protocols

# Author comment: Author response to Decision Letter 1

**DOI:** 10.1371/journal.pcbi.1014432.r002

26 Feb 2026

Attachment Submitted filename: Response to Reviewers.pdf

# Peer review: Decision Letter 1

**DOI:** 10.1371/journal.pcbi.1014432.r003

**Authors:** Gunnar Blohm, Marieke van Vugt, Gunnar Blohm, Marieke van Vugt

5 Apr 2026

PCOMPBIOL-D-25-02142R1

Spiking neurons as predictive controllers of linear systems

PLOS Computational Biology

Dear Dr. Agliati,

Thank you for submitting your manuscript to PLOS Computational Biology. After careful consideration, we feel that it has merit but does not fully meet PLOS Computational Biology's publication criteria as it currently stands. Therefore, we invite you to submit a revised version of the manuscript that addresses the points raised during the review process.

Please submit your revised manuscript by Jun 05 2026 11:59PM. If you will need more time than this to complete your revisions, please reply to this message or contact the journal office at ploscompbiol@plos.org. When you're ready to submit your revision, log on to https://www.editorialmanager.com/pcompbiol/ and select the 'Submissions Needing Revision' folder to locate your manuscript file.

Please include the following items when submitting your revised manuscript:

* A letter that responds to each point raised by the editor and reviewer(s). You should upload this letter as a separate file labeled 'Response to Reviewers'. This file does not need to include responses to formatting updates and technical items listed in the 'Journal Requirements' section below.

* A marked-up copy of your manuscript that highlights changes made to the original version. You should upload this as a separate file labeled 'Revised Manuscript with Track Changes'.

* An unmarked version of your revised paper without tracked changes. You should upload this as a separate file labeled 'Manuscript'.

If you would like to make changes to your financial disclosure, competing interests statement, or data availability statement, please make these updates within the submission form at the time of resubmission. Guidelines for resubmitting your figure files are available below the reviewer comments at the end of this letter.

We look forward to receiving your revised manuscript.

Kind regards,

Gunnar Blohm, Ph.D.

Academic Editor

PLOS Computational Biology

Marieke van Vugt

Section Editor

PLOS Computational Biology

**Journal Requirements:**

1) We have noticed that you have uploaded Supporting Information files, but you have not included a list of legends. Please add a full list of legends for your Supporting Information files after the references list.

Note: If the reviewer comments include a recommendation to cite specific previously published works, please review and evaluate these publications to determine whether they are relevant and should be cited. There is no requirement to cite these works unless the editor has indicated otherwise.

**Reviewers' comments:**

Reviewer's Responses to Questions

Reviewer #1: The authors have addressed my comments. There remain a point that I failed to fully understand about the fact that in the reactive case the network does not spike (Fig. 4). Apologies if it is simple but I was worried that the notions of predictive control and reactive control were misleadingly opposed. Take the stochastic optimal control formalism, it is purely reactive in the sense that it is a state feedback controller in the linear case, whereas there is some predictive scheduling of the feedback gain matrix. Could the author consider reformulate the explanation? Is it that what is called “reactive case” corresponds to f=0? In which case there is a risk that reactive be misunderstood for “feedback”, while the latter can exhibit some predictive structure.

Reviewer #2: I thank the authors for their explanations and additional simulations. The manuscript is much more readable now. I do however have some remaining comments that need to be addressed before this manuscript can be accepted:

eqn 5, also line 812 : r_i is a vector (spiketrain of neuron i), but the rest of the equation is scalar? Please explain

line 267: able to observe future states -> predict future states

line 312: "The local nature of the spiking rule helps with preventing this noisy spiking to affect the

resulting control performance." How do you know it's the local nature? Also, could you please specify the noise you injected and the limits where performance breaks down?

Fig. 6: "As described in S1 Appendix, the measure of acceleration is chosen for simplicity of interpretation in our framework. However, measures of work done on the plant can also be adopted to describe the energy input" What is the 'simplicity of interpretation'? I propose to call this quantity 'integrated acceleration' instead

line 354 ff: for the predictive control, you need a state predictor. What system is doing the predicting? Is that spiking too? Please comment.

line 388: "With an adequate future time window f" what is that? Can it be related to the dynamical parameters of the system (spring constant vs. dampening)? if this approach is going to be useful, knowing how to choose f is important. Please explain

line 483 ff, arm reaching task. What were the parameters used here? Is this predictive control? How long was the f window? How does the arm respond to unexpected perturbations on the way to the target?

Notebooks:

- README: 'control' is also a required library

- fig 6 is still empty for me (simulations run, but plot shows nothing)

- in fig 9:

plt.savefig('figs/fig11/axBfig11.svg', dpi=300) should this be renamed to fig9 ?

in fig 10:

line 26 Bk = np.zeros((2*M, N)) should this be Nk ?

plt.savefig('figs/fig9.png', dpi=300) should this be fig10 ?

in fig 7,11: figure 11 is saved as figure 10

**********

**Have the authors made all data and (if applicable) computational code underlying the findings in their manuscript fully available?**

The PLOS Data policy requires authors to make all data and code underlying the findings described in their manuscript fully available without restriction, with rare exception (please refer to the Data Availability Statement in the manuscript PDF file). The data and code should be provided as part of the manuscript or its supporting information, or deposited to a public repository. For example, in addition to summary statistics, the data points behind means, medians and variance measures should be available. If there are restrictions on publicly sharing data or code —e.g. participant privacy or use of data from a third party—those must be specified.

Reviewer #1: Yes

Reviewer #2: Yes

**********

PLOS authors have the option to publish the peer review history of their article (what does this mean?). If published, this will include your full peer review and any attached files.

If you choose “no”, your identity will remain anonymous but your review may still be made public.

**Do you want your identity to be public for this peer review?**  For information about this choice, including consent withdrawal, please see our Privacy Policy.

Reviewer #1: No

Reviewer #2: **Yes:** Dominik Endres

[NOTE: If reviewer comments were submitted as an attachment file, they will be attached to this email and accessible via the submission site. Please log into your account, locate the manuscript record, and check for the action link "View Attachments". If this link does not appear, there are no attachment files.]

**Figure resubmission:**

-->While revising your submission, we strongly recommend that you use PLOS’s NAAS tool (https://ngplosjournals.pagemajik.ai/artanalysis) to test your figure files. NAAS can convert your figure files to the TIFF file type and meet basic requirements (such as print size, resolution), or provide you with a report on issues that do not meet our requirements and that NAAS cannot fix.

After uploading your figures to PLOS’s NAAS tool - https://ngplosjournals.pagemajik.ai/artanalysis, NAAS will process the files provided and display the results in the "Uploaded Files" section of the page as the processing is complete. If the uploaded figures meet our requirements (or NAAS is able to fix the files to meet our requirements), the figure will be marked as "fixed" above. If NAAS is unable to fix the files, a red "failed" label will appear above. When NAAS has confirmed that the figure files meet our requirements, please download the file via the download option, and include these NAAS processed figure files when submitting your revised manuscript.

**Reproducibility:**

To enhance the reproducibility of your results, we recommend that authors of applicable studies deposit laboratory protocols in protocols.io, where a protocol can be assigned its own identifier (DOI) such that it can be cited independently in the future. Additionally, PLOS ONE offers an option to publish peer-reviewed clinical study protocols. Read more information on sharing protocols at https://plos.org/protocols?utm_medium=editorial-email&utm_source=authorletters&utm_campaign=protocols

# Author comment: Author response to Decision Letter 2

**DOI:** 10.1371/journal.pcbi.1014432.r004

3 Jun 2026

Attachment Submitted filename: Response_to_reviewers.pdf

# Editor report: Decision Letter 2

**DOI:** 10.1371/journal.pcbi.1014432.r005

**Authors:** Gunnar Blohm, Marieke van Vugt, Gunnar Blohm, Marieke van Vugt, Gunnar Blohm, Marieke van Vugt

11 Jun 2026

Dear Mr. Agliati,

We are pleased to inform you that your manuscript 'Spiking neurons as predictive controllers of linear systems' has been provisionally accepted for publication in PLOS Computational Biology.

Before your manuscript can be formally accepted you will need to complete some formatting changes, which you will receive in a follow up email. A member of our team will be in touch with a set of requests.

Please note that your manuscript will not be scheduled for publication until you have made the required changes, so a swift response is appreciated.

IMPORTANT: The editorial review process is now complete. PLOS will only permit corrections to spelling, formatting or significant scientific errors from this point onwards. Requests for major changes, or any which affect the scientific understanding of your work, will cause delays to the publication date of your manuscript.

Should you, your institution's press office or the journal office choose to press release your paper, you will automatically be opted out of early publication. We ask that you notify us now if you or your institution is planning to press release the article. All press must be co-ordinated with PLOS.

Thank you again for supporting Open Access publishing; we are looking forward to publishing your work in PLOS Computational Biology.

Best regards,

Gunnar Blohm, Ph.D.

Academic Editor

PLOS Computational Biology

Marieke van Vugt

Section Editor

PLOS Computational Biology

***********************************************************

# Editor report: Acceptance letter

**DOI:** 10.1371/journal.pcbi.1014432.r006

**Authors:** Gunnar Blohm, Marieke van Vugt, Gunnar Blohm, Marieke van Vugt, Gunnar Blohm, Marieke van Vugt

PCOMPBIOL-D-25-02142R2

Spiking neurons as predictive controllers of linear systems

Dear Dr Agliati,

I am pleased to inform you that your manuscript has been formally accepted for publication in PLOS Computational Biology. Your manuscript is now with our production department and you will be notified of the publication date in due course.

The corresponding author will soon be receiving a typeset proof for review, to ensure errors have not been introduced during production. Please review the PDF proof of your manuscript carefully, as this is the last chance to correct any errors. Please note that major changes, or those which affect the scientific understanding of the work, will likely cause delays to the publication date of your manuscript.

Soon after your final files are uploaded, unless you have opted out, the early version of your manuscript will be published online. The date of the early version will be your article's publication date. The final article will be published to the same URL, and all versions of the paper will be accessible to readers.

For Research, Software, and Methods articles, you will receive an invoice from PLOS for your publication fee after your manuscript has reached the completed accept phase. If you receive an email requesting payment before acceptance or for any other service, this may be a phishing scheme. Learn how to identify phishing emails and protect your accounts at https://explore.plos.org/phishing.

Thank you again for supporting PLOS Computational Biology and open-access publishing. We are looking forward to publishing your work!

With kind regards,

Sharmila Kamatchi

PLOS Computational Biology | Carlyle House, Carlyle Road, Cambridge CB4 3DN | United Kingdom ploscompbiol@plos.org | Phone +44 (0) 1223-442824 | ploscompbiol.org | @PLOSCompBiol
